# Circulating mouse Flk1+/c-Kit+/CD45- cells function as endothelial progenitors cells (EPCs) and stimulate the growth of human tumor xenografts

**DOI:** 10.1186/1476-4598-13-177

**Published:** 2014-07-22

**Authors:** Jeffery S Russell, J Martin Brown

**Affiliations:** 1 Head and Neck/Endocrine Oncology, Moffitt Cancer Center, 12902 Magnolia Drive, Tampa, FL 33612, USA; 2 Division of Radiation and Cancer Biology, Department of Radiation Oncology, Stanford University School of Medicine, 1050A Arastradero Rd., Rm A246, Palo Alto, CA 94304-1334, USA

**Keywords:** Vasculogenesis, Endothelial progenitor cell, Angiogenesis, Tumor, Microenvironment, Therapy, Resistance

## Abstract

**Background:**

Endothelial progenitor cells (EPCs) have been demonstrated to have stem-cell like as well as mature endothelial functions. However, controversy remains as to their origins, immunophenotypic markings, and contribution to the tumor vascular network and tumor survival.

**Methods:**

Flow cytometric analysis and sorting was used to isolate Flk-1+/c-Kit+/CD45- cells. Matrigel and methycellulose assays, flow cytometry, and gene array analyses were performed to characterize several murine EPC cell populations. Human tumor xenografts were used to evaluate the impact of EPCs on tumor growth and vascular development.

**Results:**

Flk-1+/c-Kit+/CD45- cells were present at low levels in most murine organs with the highest levels in adipose, aorta/vena cava, and lung tissues. Flk-1+/c-Kit+/CD45- cells demonstrated stem cell qualities through colony forming assays and mature endothelial function by expression of CD31, uptake of acLDL, and vascular structure formation in matrigel. High passage EPCs grown *in vitro* became more differentiated and lost stem-cell markers. EPCs were found to have hemangioblastic properties as demonstrated by the ability to rescue mice given whole body radiation. Systemic injection of EPCs increased the growth of human xenograft tumors and vessel density.

**Conclusions:**

Flk-1+/C-Kit+/CD45- cells function as endothelial progenitor cells. EPCs are resident in most murine tissue types and localize to human tumor xenografts. Furthermore, the EPC population demonstrates stem-cell and mature endothelial functions and promoted the growth of tumors through enhanced vascular network formation. Given the involvement of EPCs in tumor development, this unique host-derived population may be an additional target to consider for anti-neoplastic therapy.

## Background

In 1997, Asahara and colleagues identified a monocytic population of adult human CD34+ cells that demonstrated clonogenicity as well as contributed to neovascularization within ischemic areas [1]. Similarly, Gill et al. demonstrated the rapid mobilization of Flk-1+/AC133+ cells into the peripheral blood after vascular trauma which coated artificial blood vessel grafts [2]. These results suggested the presence of a circulating endothelial progenitor cell (EPC) population with both stem-cell like qualities and mature endothelial function. While angiogenesis requires the sprouting and proliferation of local endothelial cells, vasculogenesis is the *de novo* formation of blood vessels from circulating endothelial precursor cells. EPCs are thought to be recruited through the circulation by an incompletely defined cytokine-mediated pathway to sites of vascular injury or hypoxia. In addition to self-renewal, EPCs differentiate into mature endothelial cells and release proangiogenic cytokines and growth factors in order to form new blood vessels and/or incorporate into existing vasculature [3-5].

The potential for adult peripheral blood to contain a cellular subpopulation with the ability to repair damaged vasculature has generated intense interest in this field. Patients with pathological disorders such as stroke, heart disease, peripheral vascular disease, myocardial infarction, pulmonary diseases, and potentially the many complications of diabetes could benefit from a renewable cell population that repairs damaged vasculature [6-12]. However, malignant tumors may exploit these “beneficial” EPCs in order to obtain oxygen, growth factors and other nutrients, expand the tumor vasculature as well as to provide access to other sites of growth, resulting in metastatic spread of the disease [13-15]. Thus, vascular recovery via a circulating EPC mechanism may be a parallel or backup pathway to the well-defined angiogenesis pathway [3,16,17]. The existence of a secondary network for tumor blood vessel generation and/or maintenance may be partially responsible for resistance mechanisms to anti-neoplastic therapies and the limited clinical benefit seen using anti-angiogenic inhibitors [18-21].

Unfortunately, even with a decade and a half a research there remains significant controversy with regard to EPCs as well as many unanswered questions [13,22-26]. First, which immunophenotypic markers define this population? Second, what is the origin of these cells and how are they recruited to areas of vascular damage? And finally, with respect to oncology, what is the contribution of endothelial progenitor cells to tumor vascular networks and tumor growth and how might this affect resistance to anti-cancer therapies?

We have selected immunophenotypic markers to define a cell population that was not of hematopoietic origin (CD45 negative), but would demonstrate endothelial features (Flk-1/VEGFR-2+) as well as a stem cell marker (c-Kit+). Prior reports have suggested that this core phenotype (endothelial marker, stem cell marker, and not derived from hematopoietic cell lines) is able to select for EPCs [27-31]. Our goal in this study was to identify a population of EPCs in a murine model and to manipulate this population using *ex vivo* techniques to characterize their function. Additionally, we wanted to determine if EPCs were present in *in vivo* human tumor xenografts and to investigate their role in tumor growth and tumor vascularization. Finally, we have made several novel observations with regards to EPCs, including: the wide spread distribution of EPCs in a variety of mouse organs, established *ex vivo* culture conditions for EPCs, determined that EPC localization to solid tumors is independent of tumor type, and that Flk-1+/c-Kit+/CD45- cells can rescue lethally irradiated animals.

## Results

### Isolation of Flk-1+/c-Kit+/CD45- EPC Cells

**Figure 1:**
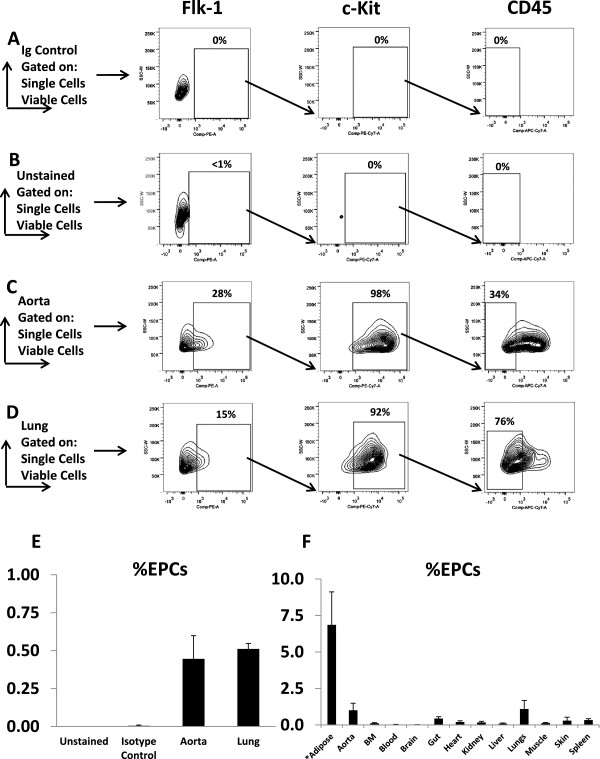
**Flow cytometry on normal nude mouse tissues after gating for viable, single cells and the sequential isolation of Flk-1+/c-Kit+/CD45- cells.** Representative images for cell isolation: **(A)** Unstained cells **(B)** Isotype controls **(C)** Isolated Aorta **(D)** Isolated Lung **(E)** Quantification of %EPCs found by flow cytometry of mouse aorta and lung tissues (n=3) **(F)** Quantification of %EPCs found by flow cytometry in selected mouse tissues (n=3-4) *Note: adipose tissue was collected from 3 different mice to generate a single sample (50,000 cells) and 3 separate samples were analyzed.

Mouse tissue were isolated and homogenized to generate a single cell suspension as described. Cells were analyzed by flow cytometry by first gating on single cells and then on viable cells. Samples were then processed in a sequential manner to isolate cells that were positive for the endothelial marker Flk-1 (VEGFR2), then for the stem cell marker c-Kit positive fraction. The dual population of Flk-1+/c-Kit+ cells was then analyzed for CD45 expression and only those cells that were CD45 negative were selected for further assays. Figure 1A and B demonstrate isolation of unstained mouse aorta/vena cava suspensions and isotype control samples which do not show any viable populations of Flk-1+/c-Kit+/CD45- cells, as expected. Figure 1C demonstrates mouse aorta/vena cava samples with a small “tail” population positive for Flk-1. The Flk-1 positive fraction of cells demonstrated higher levels of c-Kit + staining, and when this subset of cells was analyzed for CD45, slightly less than half the cells were CD45 negative. Similar results were obtained for normal mouse lung tissue, Figure 1D. Overall, the Flk-1+/c-Kit+/CD45- cells made up a very small population of the total number of cells analyzed from the tissue; as seen in Figure 1E, quantification of this population showed it to be approximately 0.5% of the total cellular population. Given the small quantities of cells isolated from the mouse aorta/vena cava and lung samples, we screened a wide variety of mouse tissues in order to determine which organs would provide the highest yield of cells. Figure 1F shows that the adipose, aorta/vena cava and lung samples were the highest yielding tissues. Tissue such as brain, liver, muscle, and blood had the lowest levels of Flk-1+/c-Kit+/CD45- cells. Interestingly, the collected mouse adipose tissue had the highest level of Flk-1+/c-Kit+/CD45- cells found; however, subcutaneous fat was difficult to obtain from the young nude mice used in this study and fat collections from 3 different nude mice were used to generate one “sample” of fat tissue and three samples were then used for analysis. Furthermore, only 50,000 cells could be isolated from this conglomerate adipose tissue sample for FACS analysis in contrast to the 100,000 cells readily available from the other tissues.

**Figure 2:**
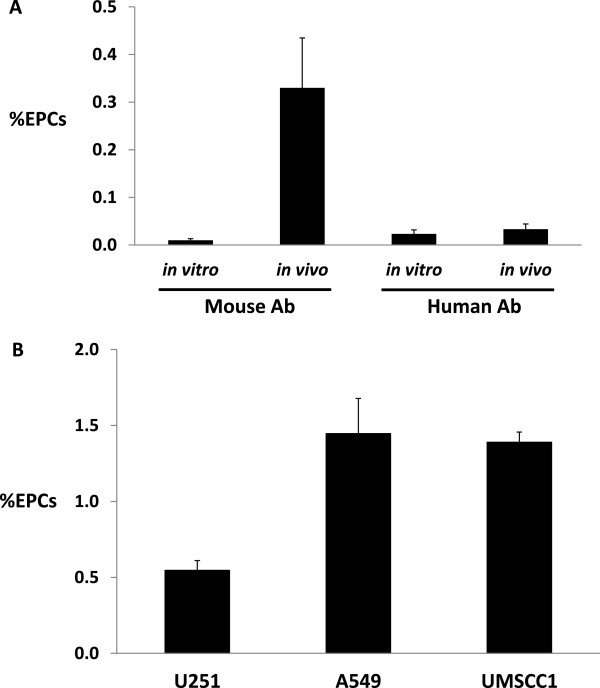
**%EPCs in tumor xenografts: (A) %EPCs isolated from U251 cells *in vitro * and *in vivo * tumor xenografts samples and reactivity based on antibody source (n=3) (B) %EPCs harvested from human tumor cell line xenografts (U251 Glioma, A549 Lung, and UMSCC1 head and neck cancer) samples (n=3).**

After the identification of a small resident population of Flk-1+/c-Kit+/CD45- cells within most mouse organs, we determine whether human tumor xenografts also had baseline levels of this cellular subpopulation. A similar FACS analysis was performed on several human tumor xenografts. As shown in Figure 2A, *in vitro* samples of U251 human glioma cells were not reactive to either anti-mouse or anti-human antibodies. However, using *in vivo* U251 tumors, mouse antibodies directed against Flk-1+/c-Kit+/CD45- populations found baseline levels of approximately 0.35%. Anti-human antibodies directed against the same markers did not react with any cells, indicating that the population of interest was derived from the host animal and not derived from the tumor cells themselves. Further experiments (Figure 2B) demonstrated increased levels of the selected EPC populations of cells in A549 lung carcinoma and UMSCC-1 head and neck carcinoma cell line at approximately 1.45% and 1.40%, respectively, compared to approximately 0.50% found in the U251 human glioma cell lines. This result suggested that the resident population of Flk-1+/c-Kit+/CD45- cells is tumor type independent.

### Characterization of Flk1+/c-Kit/CD45- EPC cells

**Figure 3:**
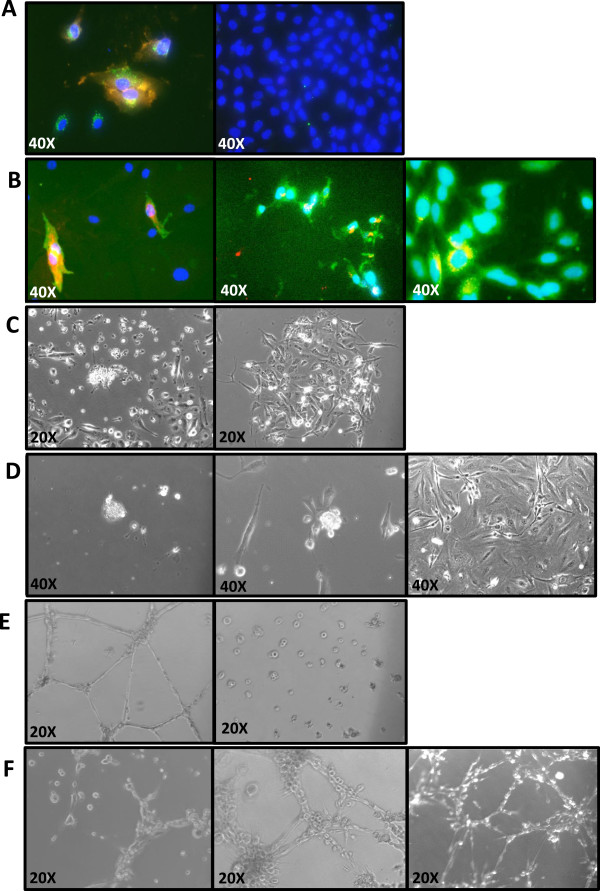
**Functional characteristics of isolated EPC cell lines. (A)** Acetylated LDL uptake in control cell lines: panel 1) HUVECs, panel 2) A549 Lung; red – CD31, green – acLDL **(B)** Acetylated LDL uptake in insolated EPC cell lines: panel 1) EPC, panel 2) EPC_acLDL, panel 3) EPC_late; red – acLDL, green – CD31 **(C)** Colony forming assay in methylcellulose with control cell lines: panel 1) mouse bone marrow samples, panel 2) A549 Lung cancer **(D)** Colony forming assay in methylcellulose with isolated EPC cell lines: panel 1) EPC, panel 2) EPC_acLDL, panel 3) EPC_late **(E)** Matrigel assay with control cell lines: panel 1) HUVECs, panel 2) A549 **(F)** Matrigel assay with isolated EPC cell lines: panel 1) EPC, panel 2) EPC_acLDL, panel 3) EPC_late.

We performed additional studies to determine if the selected subpopulation of Flk-1+/c-Kit+/CD45- cells demonstrated characteristics consistent with endothelial progenitor cells. Figure 3A (panel 1) demonstrates the membrane labeling of CD31, a known extracellular membrane marker of mature endothelial cells, as well as the peri-nuclear uptake of acetylated low density lipoprotein (acLDL), which is readily taken up by the HUVECs endothelial cell control line. Figure 3A (panel 2) demonstrates that the tumor cell line A549 does not show the CD31 membrane marker or uptake of acLDL. From our flow cytometry studies we were able to isolate the mouse lung tissue Flk-1+/c-Kit+/CD45- cell line and grow them in culture. This initial population was referred to as the EPC cell line. As seen in Figure 3B (panel 1), only about 10-20% of these cells have the CD31 marker and acLDL dual-labeling. As the acLDL fluorescent marker is non-toxic to cells, we took the acLDL-labeled EPC cell line, trypsinized the cells, and re-analyzed them by flow cytometry to select for an enriched population of acLDL + cells. As seen in Figure 3B (panel 2), the majority of cells have dual staining for CD31 and acLDL. This enriched population is referred to as the EPC_acLDL cell line. Finally, when the EPC_acLDL cell line approached passages 16–20, the morphology of the cells changed to a more flat appearance, but the cell membranes still labeled with CD31 and continued to demonstrate peri-nuclear uptake of acLDL (Figure 3B, panel 3). We refer to this high passage cell line as the EPC_Late cell line. Thus all three isolated EPC cell lines demonstrated CD31+ staining and uptake of acLDL, both known characteristics of mature endothelial cells.Additionally, we also sought to determine if our isolated cells lines demonstrated stem-cell like characteristics suggestive of self-replication. Figure 3C (panel 1) demonstrates that isolated mouse bone marrow grown in methylcellulose media had colony forming ability. In contrast, Figure 3C (panel 2) demonstrates the A549 tumor cell line generates a typical flat growth morphology, but does not generate the cluster-like cell formations seen in the control mouse bone marrow samples. Our generated cell lines (EPC and EPC_acLDL) demonstrate cell-cluster formations consistent with the ability to form colonies (Figure 3D, panel 1 and panel 2, respectively). Interestingly, the EPC_Late cell line demonstrated only the flat growth morphology with no cluster-like formations (Figure 3D, panel 3). Additionally, stem cell marker studies using alkaline phosphatase analysis and Oct-1 immunofluorescence were negative in all three cell lines (data not shown). Finally, a classic marker of endothelial function is the ability to form capillary-like structures when grown on matrigel coated plates. Figure 3E demonstrates the formation of capillary-like structures from HUVECs (panel 1) and lack of these structures in the A549 cell line when grown on matrigel. All three of our isolated cell lines (EPC, EPC_acLDL, and EPC_Late) were all able to generate capillary-like structures on matrigel plates.

**Figure 4:**
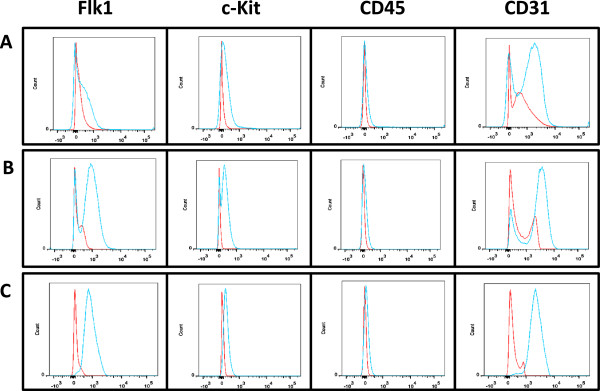
**Immunophenotype markers on sorted mouse EPCs after culturing *in vitro*: (A) EPC, (B) EPC_acLDL, (C) EPC_late. Red, isotype control’ Blue, samples.**

After generating the three cell lines as above, we then checked the cell lines for the original markers they were selected for to determine if these markers stayed intact *in vitro* or changed over time. Figure 4A demonstrates that the EPC cell line had low levels of Flk-1 expression, low levels of c-Kit expression, no significant CD45 expression, and high levels of CD31 expression. The enriched population, EPC_acLDL, demonstrated increased Flk-1 expression, enhanced c-Kit expression, no significant CD45 expression, but maintained high CD31 expression. Finally, our high passage population, EPC_Late, demonstrated Flk-1 expression, lower levels of c-Kit, no significant CD45 expression, and also maintained CD31 expression. These results suggest that the EPC_acLDL cell line is enriched for EPC functionality and that the EPC_Late population has a more mature endothelial phenotype, but may have less stem-cell like properties.

### EPCs demonstrate hemangioblastic function

**Figure 5:**
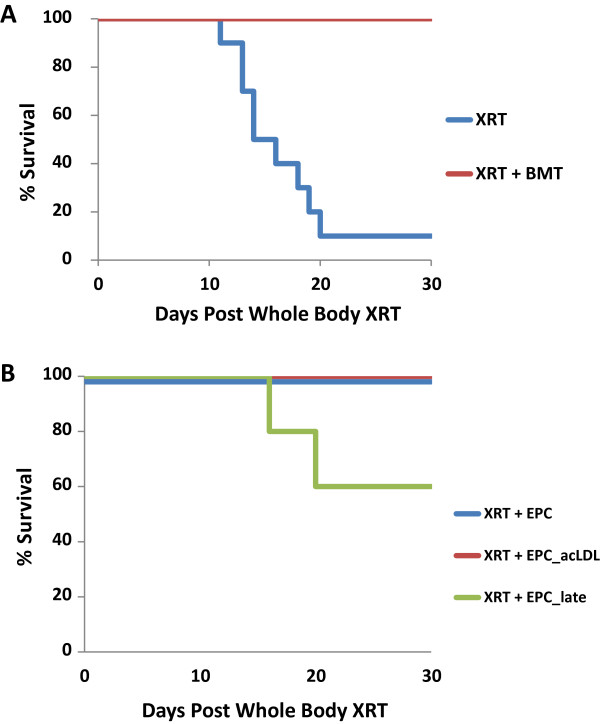
**Mouse survival after whole body radiation: (A) Survival after 9 Gy whole body radiation of nude mice with and without bone marrow transplant (n=10, each arm) (B) Survival after 9 Gy whole body radiation of nude mice with systemic transplant of EPC, EPC_acLDL, and EPC_late cell lines (n=10, each arm).**

EPC are considered to give rise to mature endothelial cells but also maintain the ability to self-replicate. However, EPCs are assumed to be downstream of a hemangioblast cell line and should be independent of hematopoietic cell lines. Thus, we undertook a negative study of our EPC cell lines to determine if they had the ability to repopulate the hematopoietic cell lines after lethal whole body irradiation. In Figure 5A, 9 Gy of whole body radiation (2 × 4.5 Gy, given 4 h apart) resulted in over 90% animal death on day 20. However, if normal healthy marrow (3×10^6^ cells) was transplanted by retro-orbital injection 2 hrs after the final dose of radiation, then 100% survival was achieved on day 20. Our hypothesis was that the isolated EPC cell lines would not be able to restore hematopoietic function; however, Figure 5B demonstrates that transplantation of 3×10^6^ cells of either the EPC cell line or the EPC_acLDL cell line were able to protect the animals from death and restore hematopoiesis. Interestingly, the EPC_Late cell line was only able to keep approximately 60% of the animals alive after whole body radiation, consistent with the above data that the EPC_Late cell line lacks some of the stem-cell characteristics demonstrated by the other isolated EPC cell lines.

### EPCs genetic similarity

**Figure 6:**
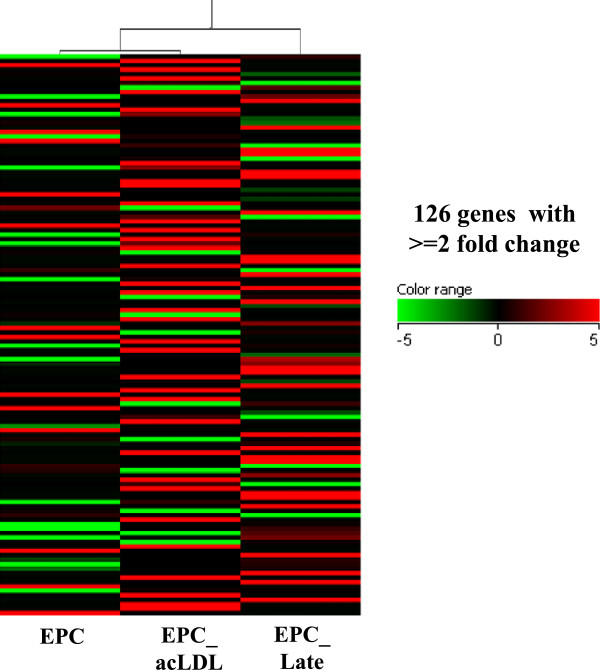
**Gene arrays: Heat map for self-organizing clusters for isolated EPC (passage 3-6), EPC_acLDL (passage 3-6), and EPC_late cell lines (passage 16-20). (3 samples each, performed in duplicate).**

In order to compare our isolated cell lines genetically, we performed a single-bead gene array analysis (Illumina, San Diego, CA) on mouse reference slides and found only 126 genes differentially regulated between the three cell lines (Figure 6A). Clustering studies showed that the EPC and EPC_acLDL cell lines appeared more closely related than the high passage EPC_Late cell line. A full list of genes up and down regulated between the cell lines is presented in Table 1. One gene of potential interest, Sox2 - often associated with stemness, was down regulated in the EPC_acLDL (3.6 fold) and EPC_Late (4.7 fold) samples as compared to the EPC parental cell line. A similar set of genes were up and down regulated between the EPC and EPC_acLDL cell lines and a greater variety of genes were changed in the EPC_Late cell line versus the EPC parental cell line. These data indicate a close relationship between all three cell lines and that the EPC_Late population is a more mature cell line with less stem cell-like characteristics.

**Table 1:** Gene array results of EPC cell lines

**EPC_acLDL v EPC**		**EPC_late v EPC**		**EPC_Late v EPC_acLDL**	
**Up**	**Down**	**Up**	**Down**	**Up**	**Down**
LOC100041835	Ppa2	LOC100041835	Gadd45a	Cldn1	Idh3b
Trim41	Abcb9	LOC100044129	BC038613	Olfr884	Bcl6
Gch1	Olfr1353	H3f3a	Krt8	Zdhhc9	Nudcd1
Aldh5a1	Ubxn11	Pcyt1a	Gbp2	LOC100044129	Cldn16
Mcpt8	Prl8a9	Zdhhc9	Ubxn11	Myot	Zfp93
Zfp93	Lhcgr	Cldn1	Bcl6	Prl8a9	Olfr554
Slc19a1	Vwa2	Mcpt8	Snapc4	Vwa2	Fhod1
Dok5	Ccl27	Slc19a1	5430407P10Rik	Ccl27	Gch1
Olfr554	Cml2	Dok5	Cldn16	Cml2	Cyp2c65
Th	Actr3	Olfr884	Olfr1353	Cd209e	Mageb1
Acss2	Cd209e	Myot	Lhcgr	Ran	Atmin
Idh3b	Ran	Egfl7	Fhod1	Mobkl2a	Aldh5a1
Egfl7	Mobkl2a	EG331493	Lrrc50	H3f3a	Prss29
Nudcd1	Rxfp1	LOC100047837	Cyp2c65	Fut11	Rdh20
EG331493	Gm1961	Ntrk3	Mageb1	Rxfp1	Mink1
LOC100047837	LOC100047339	Fut11	Cdipt	LOC100047339	Moxd1
Ntrk3	Hsd17b12	1700001O22Rik	Abcb9	Hsd17b12	Olfr1276
1700001O22Rik	Nr2f6	Slc2a1	Atmin	1300017J02Rik	Klk13
Mybl1	1300017J02Rik	Slc15a2	Prss29	Slc11a1	Acss2
Slc2a1	5430407P10Rik	Vapb	Rdh20	Fusip1	Men1
Trim26	Slc11a1	Suv39h2	Mink1	Tmem43	Gstm1
Slc15a2	Fusip1	Nab1	Moxd1	1810055E12Rik	Mdh1
Cyp4f16	Tmem43	2510040D07Rik	Olfr1276	Lpin1	Th
Vapb	Gbp2	Bves	Klk13	Pcyt1a	Ccr8
Nab1	1810055E12Rik	Trim41	Nr2f6	AW146020	Stk38
2510040D07Rik	Lpin1	Ctps	Men1	Slc9a1	Cyp4f16
Pcyt1a	Cdipt	Ars2	Gstm1	Olfr126	9630058J23Rik
	Snapc4	Sfrs1	Mdh1	Tbrg1	Trim41
	AW146020	Sfrs2	Ccr8	Suv39h2	Mettl6
	Lrrc50	Tyms	Stk38	Gcgr	Dpysl2
	Slc9a1	E2f1	9630058J23Rik	Fcer1a	Nbl1
	Olfr126	Pgp	Mettl6	1110001J03Rik	Defb36
	Tbrg1	Zw10	Dpysl2	Olfr1246	Trim26
	Gadd45a		Nbl1	Kif4	Serbp1
	Gcgr		Ppa2	Egf	Lef1
	Krt8		Defb36	Ctsd	Mybl1
	Fcer1a		Serbp1	Psg23	Abcb4
	1110001J03Rik		Lef1	Tnpo1	Hibadh
	Olfr1246		Actr3	Fbxo44	BC038613
	Kif4		Abcb4	Rock1	Shisa3
	Egf		Gm1961	Rpl4	Tspyl3
	Ctsd		Hibadh	EG13909	EG667977
	Psg23		Shisa3	Actr3	Gadd45a
	Tnpo1		EG667977	Gm1961	Krt8
	Fbxo44		Igsf4a	Bves	Gbp2
	Rock1		Tspyl3	Ppa2	
	Rpl4		Sox2	Ars2	
	BC038613		Csda	Ctps	
	EG13909		Idh3b	Sfrs1	
	Igsf4a		Nudcd1	E2f1	
	Sox2		Vamp2	Tyms	
	Suv39h2		Cyp4f16	Sfrs2	
	Fut11			Pgp	
	Csda			Zw10	
	Olfr884				
	Myot				

### EPCs effects on the growth of human tumor xenografts

**Figure 7:**
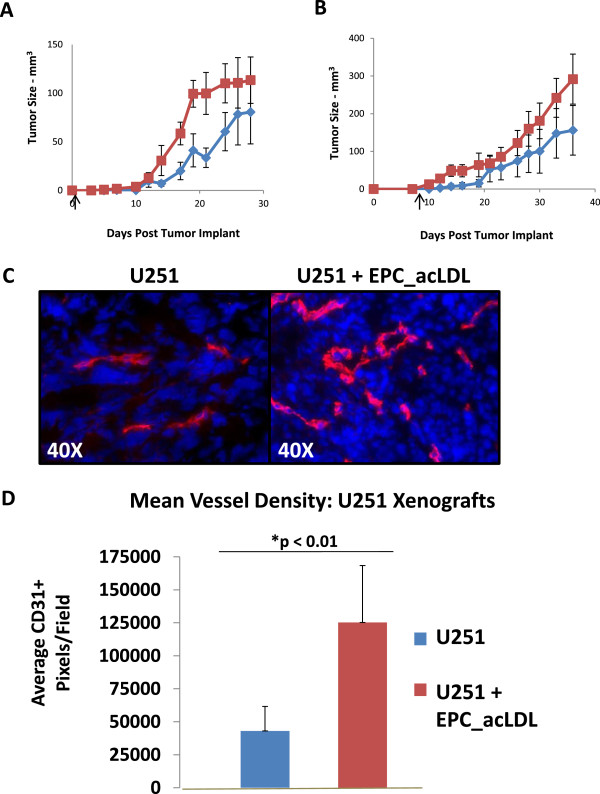
**EPC effects the vasculature in U251 xenografts. (A)** U251 Xenograft Growth Curve: Local injection of U251 only (red) or U251 tumor cells combined with EPC_acLDL cells (blue) at day 0 (arrow); Tumor Efficiency: Control 3/5 tumors; EPC_acLDL 5/5 tumors (n=5, each arm) **(B)** U251 Xenograft Growth Curve: Systemic injection (1:10) of U251 only (red) or U251 tumor cells combined with EPC_acLDL cells (blue) at day 7 (arrow); Tumor Efficiency: Control 4/5 tumors; EPC_acLDL 6/6 tumors (n=5-6, each arm) **(C)** U251 tumor xenografts with systemic injection of EPC_acLDL (1:10 ratio) collected at day 28: Representative slides stained: red, CD31; blue, Hoechst's 33258. **(D)** Quantification of Mean Vessel Density: Mean number of CD31+ pixels per 0.1 mm^2^ and standard deviation between control tumors and tumors with systemic injection of EPC_acLDL (n=3).

Finally, we wanted to determine if circulating EPCs could enhance tumor growth. First, we co-injected U251 tumor cells (3×10^6^) with the EPC_acLDL cell line (3×10^5^) at a 10:1 ratio. Figure 7A demonstrates that local co-injection of the two cell lines enhanced tumor growth and increased the efficiency of tumor take (3/5 tumors [60%] in mice with U251 cells only and 5/5 tumors [100%] in the combined U251/EPC_acLDL mixture). Thus EPC appear to enhance tumor growth in a human xenograft model. However, local co-injection does not reflect a physiological situation. Therefore, we injected the EPC_acLDL cell line (3×10^5^ cells) systemically at 7 days after the original tumor was implanted subcutaneously (3×10^6^ cells). As seen in Figure 7B, systemic injection of EPC_acLDL cells enhanced tumor growth as compared to PBS vehicle controls. To evaluate the impact of EPC_acLDL injection on vascular growth, we stained tumor samples from the growth curve samples at day 28 using CD31 as a marker of endothelial cells and blood vessels. Figure 7C demonstrates increased numbers of blood vessels in the animals with the systemic injection of EPC_acLDL cells versus control tumors. Figure 7D is the quantification of mean vessel density between the two groups. Tumor blood vessel density was significantly increased in the EPC_acLDL injected animals compared to the control animals. Vascularity was enhanced in both central tumor regions as well as at the periphery (data not shown). These data suggest that systemic injection of EPC_acLDL cells enhances tumor cell growth by increasing the tumor vascular network.

## Discussion

The selection of immunophenotypic markers of EPCs with respect to the model system used is critical [32]. While CD34 has been used previously to identify a human cell population with clonogenic potential, it can also be highly expressed on hematopoietic stem cells as well in some mature endothelial cells [33]. Similarly, the CD133 antigen has also been identified as a stem cell marker in humans, but it also commonly labels human hematopoietic stem cells and its role in a mouse model is uncertain [34]. However, the c-Kit antigen has been proposed as a murine marker of cells with clonogenic potential and to potentially identify circulating endothelial cells [35]. Recent data from Fang and colleagues, using a murine model, defined a rare population of c-Kit+ cells that were able to self-renew and found that a single, transplanted c-Kit + cell was able to proliferate and formed part of the vascular network [30]. Similarly, Fazel et al. demonstrated that c-Kit+ cells were recruited from the bone marrow to infarct border zones in cardiac tissue in a mouse model of myocardial infarction and promoted cardiac repair [36]. While CD45 is often used as a marker of true hematopoietic cells, the negative population can be used to screen out non-hematopoietic cell populations [37]. Finally, Flk-1 (VEGFR-2) is a common marker of mature endothelial cells as well as endothelial progenitor cells [33]. Based on these considerations, we isolated the Flk-1+/c-Kit+/CD45- population to capture a circulating non-hematopoietic cell population with both stem cell-like features as well as mature endothelial functions in a mouse model system. These same markers were also used by Shaked and colleagues in their studies of the mobilization and colonization of circulating endothelial progenitor cells following treatment with vascular disrupting agents and chemotherapy [17,38].

In the original studies of EPCs, circulating monocytic populations were isolated by attaching to flasks and then analyzed by flow cytometry to verify immunophenotypic markings [1]. However, using a broad monocytic population for the initial selection and growing the cells *ex vivo* in supplemental media may have generated non-physiologic conditions prior to marker analysis. This could result in the selection of a heterogeneous population with a diverse immunophenotypic expression profile that may not be reflective of the *in vivo* system. Our approach of using flow cytometry first to identify the immunophenotypic markers and then sorting viable cells to *ex vivo* culture conditions should identify an enriched, homogeneous EPC population that reflects the physiologic state of the tissue sampled. Additionally, we confirmed that isolated cells grown *ex vivo* maintained their immunophenotypic expression patterns during low passage numbers.

We found that EPCs are present in most mouse tissues, albeit at very low levels. While adipose tissue appeared to have the highest concentration of EPC at approximately 6.50%, these samples required multiple adipose tissue sites (i.e. abdominal, mesenteric, and renal fat pads) from multiple mice to generate sufficient numbers of cells to be analyzed by flow cytometry in our young mice. Other mouse organs had very low levels of EPCs (<1.0%) with samples from aorta/vena cava and lung tissue sites having the highest expression of around 0.5%. Interestingly, samples from circulating blood and brain tissue sites had the lowest levels of EPCs present. Our study only examined resident EPCs in female nude immunodeficient mice; whether these findings are similar in immunocompetent mice remains to be evaluated. While EPCs are part of a very small cell population, they could clearly be detected above background levels when compared to unstained, isotype controls. Additionally, the low levels of EPCs present in our model system is in agreement with most previously published studies [1,25,33,39,40]. An elegant study by Peters et al. found that in human patients that have undergone bone marrow transplants and subsequently developed solid tumors, bone marrow-derived cells contributed to tumor blood vessel formation at incorporation levels of around 5.0% [26]. Our data also confirmed localization of resident EPCs to a variety of tumor types and that the EPCs are derived from the host animal and are not the result of “trans-differentiation” of the implanted tumor cells.

The elevated EPC concentration in adipose tissue is also consistent with findings from other investigators. Grenier et al. isolated a tissue-resident cell population from adipose tissue that demonstrated the ability to generate stem-like spheres in culture and expressed the Sca1+ stem cell marker [41]. Additionally, as this population differentiated *in vitro*, they expressed Flk-1 and CD31, were able to take up acLDL, and enhanced vasculogenesis during muscle regeneration. A study by Yang et al. demonstrated that murine white adipose tissue contains a population of stem cells that can be recruited to tumor cells and enhance human tumor xenograft vascularization and growth [42]. Finally, Martin-Padura and colleagues demonstrated significant enrichment of CD34+/CD45- stem cells in adipose tissue over that of the bone marrow and that adipose stem cells could enhance tumor vascularization and growth in human tumor xenografts in mice [43]. One of the limitations of our study was the inability to find the correct *in vitro* culture conditions to grow the adipose-derived Flk-1+/c-Kit+/CD45- cell population in order to have stable populations to use for additional experiments. Adipose-resident EPCs could be a significant contributor to circulating EPCs (i.e. a second reservoir besides bone marrow), but additional studies with obese mouse models (to enrich the source tissue) and/or the use of fluorescently-labelled adipose tissue transplants would be required to answer these questions.

Prior studies have used colony forming and matrigel assays to define cell populations with both stem-like and mature endothelial functions. Our results demonstrate that the original Flk-1+/c-Kit+/CD45- EPC cell line as well as the enriched EPC_acLDL population maintain both of these characteristics. The high passage cell line EPC_Late appears to lose the ability to generate colonies in methylcellulose, but is able to form capillary-like structures on matrigel. This is consistent with our flow cytometry data indicating a decrease in c-Kit expression in the EPC_Late cell line. All three cell lines expressed CD31 and were able to take up the acLDL marker, indicating mature endothelial function.

With these populations established, we asked a novel question of whether these cell lines could prevent animal death in a bone marrow transplant model. We hypothesized that a true EPC population (considered to be downstream of the hemangioblast and a separate cell line from hematopoietic cells) would not be able to regenerate the hematopoietic cell lines. However, after whole body ionizing radiation, transplants of the EPC and EPC_acLDL cell lines did allow the bone marrow to recover, indicating that our EPC and EPC_acLDL populations had hematopoietic potential. Interestingly, the EPC_Late cell population was only partially able to support bone marrow recovery. Several other laboratories have also observed the effect of endothelial cells supporting the survival of bone marrow cells *in vitro*[44-47]. Chute and colleagues also demonstrated that Flk-1+ primary mouse brain endothelial cells injected systemically after whole body irradiation resulted in approximately 60% mouse survival compared to 0% of the controls and suggested that endothelial cell-derived hematopoietic activity was responsible [48]. Similarly, Montfort et al. found that transplantation of aorta or vena cava segments into lethally irradiated mice also provided a radioprotective effect [49]. At this time, it is unclear if the injected EPCs differentiate into hematopoietic cells or if perhaps cytokines produced by the EPCs can provide a protective response (“providing a vascular niche”) to bone marrow cells and/or accelerates bone marrow recovery [50]. Further studies are necessary to elucidate the mechanism(s) of bone marrow recovery.

In our genetic analysis we found that the three cell lines were quite similar with the greatest differences being between the original EPC cell line and the EPC_Late cell line and similar sets of genes were up and down regulated between the cell lines. One gene of interest that stood out was Sox2, a known stem cell marker. Sox2 was expressed at highest levels in the original cell line EPC and was down regulated in the enriched EPC_acLDL population and was further downregulated in the more differentiated cell line, EPC_Late. These studies established the close similarities of the derived cell lines; the exploration of any specific gene involvement in EPC function remains to be investigated in future studies.

To determine the impact of EPCs on tumor growth and vascularization, we demonstrated that a local injection of an EPC and tumor cell mixture as well as the systemic injection of EPCs resulted in enhanced tumor initiation and growth. The quantity of circulating EPCs is considered to be quite low, averaging <5% with most reports suggesting <2% [26,29,39]. We used a 10% ratio of the EPC_acLDL cell line to the tumor cell line to reflect the lower physiologic concentration of circulating EPCs. Martin-Padura et al. used a 20% ratio when injecting harvested CD34+ progenitors from adiopose tissue and combined with MDA-MB-436 cells and injecting into mammary fat pads [43]. Similarly, He et al. also used a ratio of 20% when injecting harvested bone marrow cells with RM1 mouse prostate cancer cells into flanks of C57BL/6 mice [51]. Thus, while our system may not reflect the ideal physiologic system, our ratio of 1:10 is consistent and slightly more restrictive than prior studies. Systemic injection of the enriched EPC_acLDL cell line significantly enhanced blood vessel formation in the tumors as demonstrated by CD31+ staining. Whether injected EPCs directly established new vasculature or contributed to vascular growth through the expression of proangiogenic cytokines will need to be addressed in future studies. In examining the contribution of circulating EPCs toward the development of tumor blood vessel, Lyden et al. demonstrated that in bone marrow ablated mice, bone marrow transplant rescued tumor angiogenesis in mice that did not express a key enzyme of endothelial cell function, nitric oxide synthase [13]. In contrast, DePalma et al. failed to find transplanted bone marrow cells expressing a labeled TIE-2 promoter within tumor vasculature [39]. Similarly, Purhonen et al. used a labeled parabiosis mouse model to establish collateral circulation but failed to find labeled bone marrow-derived cells in the partner mouse tumor vasculature, though a few bone marrow-derived cells were present in the perivascular niche [29]. However, several additional studies have found that tumor grade, tumor type, as well as the tumor site of origin may regulate the incorporation of circulating EPCs into tumor-associated blood vessels [52-54].

Finding a specific cell type, such as EPCs, that directly influences tumor growth and vascularization may have a substantial clinical impact. These cells may influence tumor resistance to chemotherapy or radiation treatment and may help promote tumor recovery after surgical resection. Thus, understanding EPCs will provide benefit as a possible therapeutic target. However, prior EPCs studies have presented a spectrum of conflicting results. In our study we have focused on circulating Flk-1+/c-Kit+/CD45- which have clearly demonstrated EPC function. We acknowledge that there may be alternative immunophenotypic markers that define other cell populations with EPC functionality. We have further established that the Flk-1+/c-Kit+/CD45- population, even at the low level of circulating cells identified in this study and others, can play a significant role in tumor growth and tumor vascularization. Additionally, a novel property found in this study was the hemangioblastic potential of the Flk-1+/c-Kit+/CD45- cell population and this ability could significantly impact tumor survival. The full contribution of this finding remains to be elucidated. Given the potential for EPCs to support tumor growth, they may also contribute to the resistance of tumors during treatment. Additional studies are ongoing to address the contribution of EPCs to resistance mechanisms as well as the consideration of an anti-EPC strategy to enhance current anti-neoplastic therapies.

## Conclusions

In summary, our current study has identified Flk-1+/C-Kit+/CD45- cells as endothelial progenitor cells with both endothelial and stem-like qualities. Furthermore, circulating EPCs enhance tumor initiation, tumor growth, and increase tumor vascularity. Future studies will investigate the origins of EPCs and determine their role in mediating resistance to anti-cancer therapies.

## Materials and methods

### Antibodies and reagents

Selected antibodies were acquired as indicated: Rat IgG Isotype control (553993, BD Pharmingen), Rat IgG Fluorescent Isotype controls (sc-2831, sc-3788, sc-2895, sc 2872; Santa Cruz Biotechnology), Rat Anti-Mouse Flk-1-PE (555308, BD Pharmingen), Rat Anti-Mouse c-Kit-PE-Cy7 (558163, BD Pharmingen), Rat Anti-Mouse CD45-APC-Cy7 (557659, BD Pharmingen), Rat Anti-Mouse CD31-FITC (553372, BD Pharmingen), Mouse Anti-Human VEGFR2-PE (560872, BD Pharmingen), Mouse Anti-Human CD117-APC (561118, BD Pharmingen), Mouse Anti-Human CD45-APC-Cy7 (557833, BD Pharmingen), Mouse Anti-Human CD31-FITC (560984, BD Pharmingen), Rat Anti-Mouse CD31 (550274, BD Pharmingen), Goat Anti-Rat FITC (sc-2011, Santa Cruz Biotechnology), Goat Anti-Rat AlexaFluor594 (A-11007, Life Technologies), Anti-Rabbit Oct-4 (AB3209, EMD Millipore), and Goat Anti-Rabbit FITC (sc-2012, Santa Cruz Biotechnology). Additional reagents used: Hoechst’s 33258 Stain (94403, Sigma-Aldrich), acLDL-Dil (L3484, Life Technologies), acLDL-BODIPY (L3485, Life Technologies), acLDL (L35354, Life Technologies), Fibronectin (F1141, Sigma-Aldrich), and Alkaline Phosphatase Stain (A14353, Life Technologies).

### Cell lines

HUVECs were grown in human endothelial growth media with supplementation (PM211500, Genlantis, San Diego, CA). The tumor cell lines: A549 lung carcinoma, U251 glioblastoma, and UMSCC-1 head and neck squamous cell carcinoma were grown in DMEM supplemented with 10% inactivated fetal bovine serum (Life Technology) and 1% penicillin-streptomycin. All cell lines were acquired from ATCC (Manassas, VA).

### Mice

Female athymic *nu/nu* nude mice (aged 8–12 weeks) were acquired from NCI-Fredrick (Fredrick, MD). Mice were maintained in a germ-free environment and had access to food and water *ad libitum*. All animal procedures were approved by Stanford University’s Administrative Panel on Laboratory Animal Care (APLAC).

### Mouse xenografts

Tumors were implanted subcutaneously on the backs of nude mice approximately 2 cm above the base of the tail. A549, U251, and UMSCC-1 cell lines were implanted at a concentration of 3×10^6^ cells in 100 μL PBS. Tumors reached a size of approximately 200 mm^3^ in 3–4 weeks with an efficiency of approximately 90%, 70%, and 90%, respectively.

### EPCs isolation

EPC isolation was performed as reported previously with documented modifications [1,55,56]. Selected mouse tissues or tumor samples were surgically removed from euthanized nude mice. Tissues were kept in mouse endothelial media (M1168, CellBiologics, Chicago, IL) supplemented with the growth factor kit (VEGF, EGF, heparin, hydrocortisone, and L-Glumatine) and 10% fetal bovis serum (FBS) and then minced into small chunks with a #11 scalpel blade. Tissue fragments were then placed into a glass homogenizer and mechanically disrupted into cellular slurry. The slurry was transferred to 15 mL tubes and mixed with 1 mL of supplemented mouse endothelial media and combined with 2 mL of 0.1% Collagenase I (CLS-1, Worthington Biochem, Lakewood, NJ), 0.1% Collagenase IV (CLS-4, Worthington Biochem, Lakewood, NJ), and 0.001% Deoxyribonuclease I (DPRF, Worthington Biochem, Lakewood, NJ). Samples were incubated for 30 minutes at 37°C. Mouse endothelial medium was added to a total volume of 15 mL and solutions were poured through a 70 μM cell strainer into a new 15 mL tube. Tube were then spun at 4°C at 3000 rpm for 5 minutes. The supernatant was decanted and the resulting cell pellet was resuspended in 2 mL of RBC lysis buffer (1.5 M NH_4_CL, 100 mM NaHCO3, 10 mM Na_2_-EDTA; pH 7.4) and placed on ice. After 15 minutes, samples were spun down at 4°C at 3000 rpm for 5 minutes, supernatant removed, and resuspended in PBS and kept on ice. Live/Dead stain (L23105, Invitrogen) was added to the cells for 30 minutes with cells kept on ice and protected from light. Samples were then moved to eppendorf tubes and spun for 5 minutes, 4°C at 5000 rpm. Supernatant was removed and the cell pellet was washed twice in PBS, and resuspended in PBS and used for flow cytometry.

### Flow cytometry

Flow cytometry was performed in the Stanford Flow Cytometry Core Facility using the BD Aria II (sorting) or BD LSR.II (analysis). Samples were isolated from mouse tissues or tumors as above or from trypsinized from tissue culture samples. Cells were blocked with CD16/CD32 Mouse Fc Block (553142, BD Pharmingen) for 30 minutes, then labeled with anti-Flk-1:PE, anti-c-Kit:PE-Cy7, anti-CD45: APC-Cy7, and as indicated, anti-CD31: FITC. Unstained samples and samples with antibody IgG controls were run in parallel. Additionally, color compensation for each fluorochrome was evaluated using IgG compensation beads (552843, BD Pharmingen) and ArC compensation beads (A10346, Invitrogen) with appropriate fluorescent marker controls. Once lasers were tuned and color compensation was set, at least 100,000 cells per sample were run, except for the adipose tissue samples which were run at 50,000 cells per sample. Gating was initially applied to isolate single cell populations of viable cells, then additional gating was used to select for the appropriate subpopulations (Flk-1+/c-Kit+/CD45-). Flow cytometric analysis was performed using FlowJo Software (TreeStar, Inc., Ashland, OR). For the flow cytometry sorting of live cells, the same staining process was used as above. Once subpopulations of single, viable cells were gated on Flk1+, c-Kit+, and CD45- and subsequently isolated, they were then collected in an eppendorf tube with 500 μL of mouse endothelial media (M1168, Cell Biologics, Chicago, IL) supplemented with the growth factor kit (VEGF, EGF, heparin, hydrocortisone, and L-Glumatine) and 10% FBS and managed as *in vitro* cultures as described below.

### *In vitro* culturing of sorted EPCs

Sorted cell from nude mouse lung samples were placed in 5 μg/cm^2^ fibronectin treated T25 flasks in mouse endothelial medium (M1168, Cell Biologics, Chicago, IL) supplemented with the growth factor kit (VEGF, EGF, heparin, hydrocortisone, L-Glumatine, and 10% FBS) and incubated at physiologic concentrations of FiO2 (5%) at 37°C. Flasks were examined every 2–3 days and once attached cells began to generate colonies of 10–20 cells, flasks were moved to normoxic incubators (FiO2 21% at 37°C). As flasks became confluent, cells were passaged as usual. The EPC cell line and EPC_acLDL cell lines were used from passages 3–6 and the EPC_Late cell lines was used between passages 16–24.

### ICC immunofluorescence

Tumor and EPC cell lines were grown and treated in chamber slides (C1782, Sigma-Aldrich). The reagent acLDL-Dil or acLDL-BODIPY was mixed with cells at 2.5 μg/mL concentration and allowed to incubate for 4 hours at 37°C. Medium was then aspirated and cells were fixed in 4% paraformaldehyde for 10 min at room temperature. Paraformaldehyde was aspirated and the cells treated with a 0.2% NP40/PBS solution for 15 min. HUVEC cells and the human tumor cell lines used acLDL-BODIPY and the Anti-CD31-APC antibody while the murine derived EPC-related cell lines used acLDL-Dil and the Anti-CD31-FITC antibody for staining. Cells were then washed in PBS twice, and the selected anti-CD31 antibody at a dilution of 1:50 in 1% BSA was added and incubated overnight at 4°C. Cells were again washed twice in PBS before incubating in the dark with the appropriately labeled secondary antibody at a dilution of 1:100 in 1% BSA for 1 h. The secondary antibody solution was then aspirated and the cells washed twice in PBS. Cells were then incubated in the dark with Hoechst’s 33258 (1 μg/ml) in PBS for 30 min, washed twice, and coverslips mounted with an anti-fade solution (Dako Corp., Carpinteria, CA) and sealed with clear nail polish. Slides were examined on a Lecica DM6000B fluorescent microscope (Lecica, Buffalo Grove, IL). Images were captured by a CCD camera using the Image Pro Premier v9.0 (MediaCybernetics, Rockville, MD) software package.

### IHC immunofluorescence

Tissues were harvest from mice and placed in cassettes and covered in OCT media. Cassettes were held at -80°C until they were process on a cryotome and cut to 2 μm tissue sections. Slides were then kept at -80°C until processing. Slides were allowed to air dry for 10 min at room temperature and then were kept in -20°C methanol for 10 minutes. Methanol was removed and samples were allowed to air dry for 30 minutes and washed with PBS twice for 5 minutes each. The rat anti-CD31 antibody was used at a dilution of 1:20 in 2% BSA and incubated overnight at 4°C. Slides were again washed twice in PBS before incubating in the dark with a goat anti-rat AlexaFluor594-labeled secondary antibody at a dilution of 1:100 in 1% BSA for 1 h. The secondary antibody solution was then aspirated and the cells washed twice in PBS. Cells were then incubated in the dark with Hoechst’s (1 μg/ml) in PBS for 30 min, washed twice, and coverslips mounted with an anti-fade solution (Dako Corp., Carpinteria, CA) and slides were sealed with clear nail polish. Slides were examined and images captured as above.

### Colony forming assay

Selected cell lines were cultured in methylcellulose-containing medium (M3434, StemCell Technologies, Vancouver, BC, Canada) with 50 ng/mL vascular endothelial (VE) growth factor (R&D Systems, Minneapolis, MN, USA), 50 ng/mL basic fibroblast growth factor (Wako, Osaka, Japan) and 10% fetal bovine serum (FBS) on 35-mm dishes. Cell densities were 1×10^3^ for each cell lines. Dishes were plated in triplicate and kept in humidified incubator for 8–10 days until colonies started to develop. EPC colony forming units (CFUs) were defined as cluster-like collections of cells associated with attached spindle-shaped cells. The EPC-CFUs were identified by visual inspection with an inverted microscope under 20-40× magnification. Images were acquired on a Zeiss Observer. A1 (Zeiss Microscopy, Thornwood, NY) microscope using AxioVision 4.6.3 software (Zeiss Microscopy, Thornwood, NY).

### Matrigel assay

As a modified protocol of Wu et al., matrigel (356237, BD Pharmingen) was applied in thin layers to wells of 24-well plates [57]. Cells of selected cell lines were placed at indicated cellular concentrations: 1×10^4^, 1×10^5^, and 1×10^6^ cells. Cells were incubated overnight and the next morning (18 hrs) evaluated under light microscopy at 20-40X magnification. Images were acquired on a Zeiss Observer. A1 (Zeiss Microscopy, Thornwood, NY) microscope using AxioVision 4.6.3 software (Zeiss Microscopy, Thornwood, NY).

### Bone marrow transplant studies

Nude mice were exposed to two doses of 4.5 Gy given 4 hours apart and, as indicated, supplemental cells were given 2 hours after the final radiation dose [58]. Normal bone marrow was collected from unirradiated 12–16 week old mice by isolating the femurs and tibia, cutting the end from the bone, and then flushing out the central cavity using 28 gauge needles with 2% FBS in Hank’s balanced salt solution. The cell solution was collected, spun down, and cell concentration calculated. Harvested bone marrow or the selected *ex vivo* cultured EPC cell lines were injected into the retro-orbital venous plexus of an anesthetized mouse at 3×10^6^ cells in 100 uL PBS. Survival was assessed every two days and mice were sacrificed if weight had dropped more than 10% per animal protocol guidelines.

### Gene array analysis

Sorted cell lines: EPC, EPC_acLDL, and EPC_late were grown out, collected, and RNA isolated (RNeasy mini kit, Qiagen). RNA concentration and quality was measured by NanoDrop analysis. RNA was stored in RNAase free water at -80°C until analysis. RNA was hybridized to MouseRef-8 v2.0 Beadchips (25 K, Illumina, San Diego, CA). Two separate experiments were performed independently and run in duplicate at the Stanford Gene Array Core Facility. Bead level intensity values were summarized without normalization and local background correction was applied by default using Beadstudio v3.1. Microarray gene expression data were processed and analyzed using Genespring VX (Agilent, Santa Clara, CA). Clustering was performed using non-centered Pearson correlation. Data was deposited at: http://www.ncbi.nlm.gov/projects/geo (GSE53681).

### Tumor growth curves

U251 cells at a concentration of 3 × 10^6^ were mixed with 3×10^5^ EPC_acLDL cells (10% ratio). For co-injection studies, the cell mixture was implanted on the back of nude mice and tumor measurements were taken approximately three times a week. Tumor volume (mm^3^) was calculated as (height^2^*length)/2. For the systemic injection studies, Tumors (U251; 3×10^6^) were implanted on the back of mice on day 0 and EPC_acLDL cells (3 × 10^5^) were given by 100 μL retro-orbital injection on day 7 with controls receiving an injection of normal saline in an identical volume. Tumor measurements were then taken approximately three times weekly and tumor volumes calculated as above. Numeric data are presented as the mean +/- standard error.

### Mean vessel density analysis

Images from the fluorescent immunohistochemical analysis were imported into ImageJ analysis software (http://www.rsbweb.nih.gov/ij/, NIH, Bethesda, MD). A threshold of CD31 pixel intensity was applied to all images and the total number of CD31 positive pixels was counted within the field of view. Total CD31 pixel counts from five random images from each tumor sample were analyzed and averages were calculated over a 0.1 mm^2^ area [59,60]. Two tumors from two different experiments were used for analysis. Numeric data are presented as the mean +/- standard deviation.

### Statistical analysis

Statistical comparisons of datasets were performed by a two-tailed Student’s *T*-test using Microsoft Excel (Redmond, Washington). Data was considered to be significantly different when P ≤ 0.05.

## Competing interests

The authors declare they have no competing interests.

## Authors’ contributions

JSR participated in the design of the study, carried out all the experiments, drafted the manuscript, and performed statistical analysis. JMB contributed to the design of the study and editing of the manuscript. Both authors read and approved the final manuscript.
